# Application of respiratory muscle training for improved intermittent exercise performance in team sports: a narrative review

**DOI:** 10.3389/fspor.2025.1632207

**Published:** 2025-07-15

**Authors:** Tomasz Kowalski, Gabriel Dias Rodrigues, Michele Zanini

**Affiliations:** ^1^ Institute of Sport, National Research Institute, Warsaw, Poland; ^2^ Laboratory of Experimental and Applied Microbiology, Fluminense Federal University, Niterói, Brazil; ^3^ Department of Clinical and Community Sciences, Faculty of Medicine and Surgery, University of Milan, Milan, Italy; ^4^ School of Sport, Exercise and Health Sciences, Loughborough University, Loughborough, United Kingdom; ^5^ School of Education, Childhood, Youth and Sport, Faculty of Wellbeing, Education and Language Studies, The Open University, Milton Keynes, United Kingdom

**Keywords:** respiratory muscle training, repeated sprint ability, yo-yo test, football, rugby, soccer

## Abstract

Since traditional, sport-specific training or exercise programs lack sufficient stimulus to improve the function of the respiratory muscle, the rationale for integrating additional respiratory muscle training (RMT) emerged. RMT has the potential to improve intermittent exercise performance in team sports athletes, as proven in multiple studies. This narrative review aims to provide coaches with tools to select the appropriate form of RMT, tailored to the athletes’ needs, using appropriate diagnostic methods, intervention protocols, and devices. Common protocols may include performing 30 inspiratory maneuvers twice a day, five days a week, with resistance-based trainers or engaging in 20–40 min of vigorous ventilation with isocapnic devices every other day. Most of the interventions that positively influence intermittent exercise performance employed inspiratory pressure threshold loading, lasted 6–8 weeks, and relied on a high frequency of training sessions, progressive overload, and relatively high initial resistance (starting intensity). Less-investigated RMT methods, such as tapered flow resistive loading or voluntary isocapnic hyperpnea, should be analyzed in the context of intermittent exercise performance. Moreover, further research addressing RMT and hypoxia, between-gender differences, and athletes with disabilities seems warranted.

## Introduction

Team sports have gained immense popularity worldwide, involving millions of participants who engage in dynamic and physically demanding activities (1). Based on global participation, viewership, and cultural significance, disciplines such as soccer (football), basketball, cricket, rugby, hockey, baseball, volleyball, and various types of football are considered the most popular (2, 3). The performance determinants vary to a certain extent depending on the discipline and players’ specific roles (4). However, team-sports athletes are usually required to execute repeated skillful and high-intensity actions such as accelerations, changes in pace and direction, sprints, jumps, and kicks. These efforts are typically performed in cycles of maximal or near-maximal intensity, interspersed with brief recovery periods that may consist of rest or low- to moderate-intensity activity. Such activities often extend over prolonged periods, ranging from one to two hours, placing considerable demands on the athlete's physical capacities (5, 6). Noteworthy, physiological team-sports requirements are closely intertwined with the execution of specific skills, highlighting the complex and multifaceted nature of team-sport performance (7).

The combination of technical skills and physical demands requires athletes to maintain optimal conditioning tailored to the unique challenges of their sport and athlete's roles (4). The interplay between high-intensity actions and recovery periods underscores the importance of targeted physical preparation, enabling athletes to perform effectively and consistently throughout the game (8). Consequently, one of the most emphasized physical training goals is the ability to repeatedly perform intense exercise, often evaluated with the Yo-Yo Tests (YYT) or Repeated Sprint Ability (RSA) assessments (9, 10). The physiology of such testing is driven by the interplay of energy systems, muscle function, and recovery mechanisms (7, 9). RSA relies primarily on the phosphagen (ATP-PCr) system to supply rapid energy during the initial seconds of each sprint, with anaerobic glycolysis contributing as phosphocreatine (PCr) stores become depleted. These systems enable short bursts of high-intensity effort but are limited by the accumulation of metabolic by-products such as hydrogen ions, contributing to fatigue. Compared to RSA tests, which focus predominantly on anaerobic power and fatigue resistance during repeated sprints, the YYT places greater emphasis on sustained aerobic metabolism and recovery between efforts (11). In both tests, recovery between efforts depends heavily on the aerobic system, which supports PCr resynthesis, lactate clearance, and the restoration of muscle pH (7, 11, 12). Additionally, effective intermittent exercise performance requires well-developed neuromuscular efficiency and the ability to resist fatigue-related reductions in motor unit activity (13–15).

No single type of training can be universally recommended as the most effective for enhancing intermittent exercise performance or addressing all the factors contributing to output declines during repeated effort tasks (16). Typically, two recommended training goals are improved single-sprint performance and improved aerobic fitness to enhance the ability to recover between efforts (16). Noteworthy, respiratory muscle training (RMT) may improve intermittent exercise performance (17, 18). RMT is a specific conditioning method designed to enhance the strength and endurance of the muscles involved in breathing. Nicks et al. (19) and Najafi et al. (20) reported that RMT improved intermittent exercise performance in both male and female soccer players (19, 20). Tong et al. (21) noted enhanced tolerance to intense intermittent exercise after both RMT programs and respiratory muscle warm-ups (21). Romer et al. (22) also observed faster recovery time during high-intensity, intermittent exercise in repetitive-sprint athletes (22). More recently, after RMT interventions a decrease in sprint time and improved exercise tolerance during RSA assessments in professional soccer players, as well as increased distance covered by rugby athletes in YYT were reported (23, 24).

On the physiological side, RMT was reported to attenuate the blood lactate concentration, plasma ammonia, and uric acid responses during high-intensity, intermittent exercise. Moreover, it was associated with improved perceptual responses and breathlessness (21, 22). All the relevant studies included in the systematic review from Lorca-Santiago et al. reported significant decreases in perceived exertion during RSA and YYT, from 8% to 29%, with large effect sizes (18). Moreover, the mechanistic explanation may be associated with attenuated respiratory metaboreflex and improved blood flow to limb muscles during high intensity, as reported in professional women soccer players (25). Although not easy to observe in applied environments, the respiratory metaboreflex is widely associated with performance improvements originating from RMT. The practical implications stem from findings that fatigue and metabolite accumulation in respiratory muscle lead to reduced blood flow to skeletal muscles, redirecting it toward the respiratory muscle (26). This results in vasoconstriction in the active limbs during exercise, contributing to increased local fatigue and performance limitations (27). RMT enhances respiratory muscle function and is anticipated to counteract the negative effects of the metaboreflex, thereby reducing its systemic impact.

Traditional sport-specific training or exercise programs lack sufficient stimulus to improve the function of the respiratory muscles, underscoring the rationale of integrating additional RMT into training regimens (28, 29).

## Training methods and equipment

**Figure 1:**
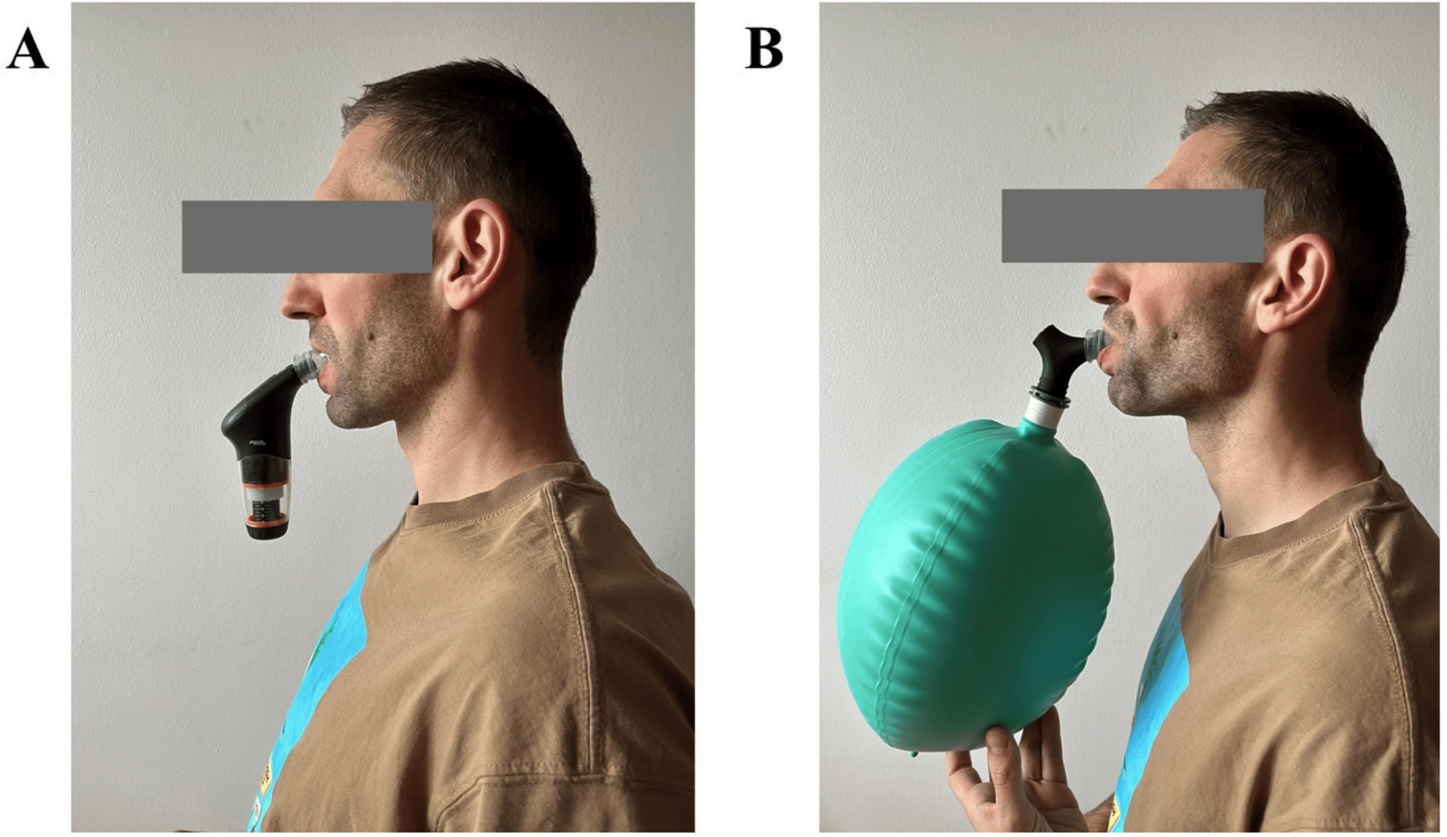
Illustrative application of two training methods. **(A)** Presentation of respiratory muscle strength training. **(B)** Presentation of respiratory muscle endurance training. Figure adapted from Kowalski et al. (30).

A wide range of RMT methods and devices are available, with three key approaches demonstrating significant benefits in athletic contexts: inspiratory pressure threshold loading (IPTL), tapered flow resistive loading (TFRL), and voluntary isocapnic hyperpnea (VIH). While TFRL and IPTL are primarily linked to enhanced respiratory muscle strength, VIH is more closely associated with improvements in respiratory muscle endurance (29). Specifically, IPTL and TFRL result in larger improvements in maximal inspiratory pressures, and VIH was associated with improved maximal voluntary ventilation, significant flow rates, and high velocities of respiratory muscle contraction (17). Illustrative application of both approaches is presented in Figure 1 (30).

TFRL and IPTL might be used as inspiratory-only, expiratory-only, or mixed respiratory muscle training. Inspiratory muscle training has consistently demonstrated benefits in improving respiratory muscle strength, endurance, and overall exercise performance in healthy and trained subjects. In contrast, evidence regarding the effectiveness of expiratory or mixed training remains inconsistent, with fewer studies addressing its potential benefits in a well-trained population. Hence, the following section focuses on inspiratory muscle training as the optimal and proven approach. IPTL and TFRL rely on dedicated breathing devices that provide resistance during inspiration and allow for expiration without additional resistance (31). Training protocols typically require individuals to perform vigorous inspirations, starting from the residual volume, against a resistance set at 30%–80% of their maximal inspiratory pressure. Most popular and studied programs require 30 quick and forceful maneuvers from functional residual capacity, twice daily for 5–6 days per week, for at least 4–6 weeks (29). A key distinction between IPTL and TFRL lies in how resistance is applied. In IPTL, the resistance remains constant throughout the breath, resulting in a progressive shift from low pressure and high airflow at smaller lung volumes to high pressure and low airflow as the lungs fill. Due to the pressure–flow dynamics of the respiratory muscle, inspiration at higher lung volumes demands greater muscle strength. Eventually, the resistance can surpass the muscle's capacity to generate sufficient inspiratory pressure, limiting further shortening of respiratory muscle and preventing full lung expansion. In contrast, TFRL features a progressively decreasing external resistance during inspiration, delivering moderate pressure and airflow evenly across the entire vital capacity range (32).

VIH involves devices equipped with partial rebreathing circuits and emphasizes controlled, intense breathing exercises. This method relies on intentional hyperpnea as the primary training stimulus, operating at an intensity of 60%–90% of maximal voluntary ventilation, with minimal or no external resistance applied. The rebreathing circuits help maintain an athlete's homeostasis, as prolonged hyperventilation without specialized equipment is not feasible and, even over short periods, can cause significant disturbances in blood gas levels and negatively impact well-being (29). VIH training programs are usually based on 3–5 sessions per week, from 15 to 40 min each, and should last at least 4–6 weeks.

Notably, not all RMT programs are associated with improved performance, as this depends on the intervention design (33, 34). Ineffective interventions may result from an insufficient training stimulus, characterized by inadequate resistance, limited program duration, or failure to implement progressive overload principles (33, 35). Moreover, low motivation and adherence to RMT programs may be significant limiting factors in achieving optimal outcomes. Consequently, providing appropriate supervision is essential to ensure consistent engagement and maximize the effectiveness of the intervention (33). On the other hand, most of the effective interventions addressing intermittent performance share common characteristics and last 6–8 weeks, employ IPTL, rely on a high frequency of training sessions, progressive overload, and relatively high initial resistance (starting intensity). A summary of protocols resulting in a significant, positive influence on intermittent exercise performance from peer-reviewed studies is presented in Table 1. Importantly, the lack of TFRL and VIH studies in Table 1 does not mean they are not effective, but understudied. The available literature that compares different RMT methods does not address intermittent exercise performance. However, studies typically report similar outcomes concerning continuous or sport-specific efforts (30, 36, 37).

**Table 1:** Summary of protocols resulting in a significant, positive influence on intermittent exercise performance from peer-reviewed studies.

Study	Population and N	Intervention characteristics	Test	Performance
Romer et al. (22)	M (24) Mixed, mostly soccer and rugby	IPTL 6 weeks 7 days/week 2 sessions/day (30 reps) 50% MIP + PO	RSA	+7%
Tong et al. (21)	M (30) Soccer and rugby	IPTL 6 weeks 6 days/week 2 sessions/day (30 reps) 50% MIP + PO	YYT	+16%
Nicks et al. (19)	M (20) F (7) Soccer	IPTL 5 weeks 5 days/week 2 sessions/day (30 reps) 50% MIP + PO	RSA	+17%
Archiza et al. (25)	F (18) Soccer	IPTL 6 weeks 5 days/week 2 sessions/day (30 reps) 50% MIP + PO	RSA	+4%–6%
Nunes Junior et al. (23)	M (20) Rugby	IPTL 12 weeks 3 sessions/week (30 reps) 80% MIP + PO	YYT	+14%
Silva et al. (24)	M (22) Soccer	IPTL 2 weeks 6 days/week 1 session/day (15–30 reps) 50% MIP	RSA	+4%–5%
Najafi et al. (20)	M (30) Soccer	IPTL 8 weeks 5 days/week 2 sessions/day (25–55 reps) 45–55% MIP + PO	YYT	+8%–9%
Antonelli et al. (58)	M (17) Wheelchair basketball	IPTL 12 weeks 5 series of 10 reps, frequency unknown 50% MIP + PO	YYT	+18%

IPTL, inspiratory pressure threshold loading; M/F, males/females; MIP, maximum inspiratory pressure; N, number of subjects; PO, progressive overload; RSA, repeated sprint ability; YYT, yo-yo test.

Products such as POWERbreathe®, Airofit, BreathWayBetter (recently released as Isocapnic), and SpiroTiger® (recently released as Idiag) are commonly used in sports science and remain most popular on the market. Their brief characteristics may be found in Table 2. Airofit devices might be particularly useful for coaches or scientists, as they offer remote supervision options, and staff may track RMT execution and progress via the online platform.

**Table 2:** Overview of RMT devices and their characteristics, prices as of June 2025.

Training equipment	Strength/endurance oriented	Inspiratory Resistance range (cmH2O)	Expiratory resistance (cmH2O)	Price (USD)	Mechanical/electronic device
POWERbreathe® K4	Strength	5–200	Not available	725	E
POWERbreathe® Plus Medium	Strength	23–186	Not available	89	M
AiroFit PRO 2.0	Strength	10–250	10–200	380	E
AiroFit Essential	Strength	20–140	30–200	249	E
SpiroTiger® Idiag P100	Endurance	Voluntary	Voluntary	1,639	E
SpiroTiger® GO	Endurance	Voluntary	Voluntary	999	E
Isocapnic BWB	Endurance	Voluntary	Voluntary	149	M

E, electronic; IPTL, inspiratory pressure threshold loading; M, mechanical; TFRL, tapered flow resistive loading; VIH, voluntary isocapnic hyperpnoea.

## Methodological considerations for optimizing RMT interventions

Training interventions should begin with a well-defined baseline, and RMT is no exception. Such an approach allows for identifying an athlete's needs and measuring adaptation or lack thereof. Therefore, an evaluation of the athlete's respiratory muscle function is recommended before introducing RMT. Noteworthy, it may be performed with easy-to-use and mobile devices, such as the above-mentioned POWERbreathe® K-Series or Airofit (38, 39).

The theory of sport outlines several key principles designed to optimize the training process and athletic performance. While different sources might list slightly different numbers or names for these principles, they are generally consistent across disciplines and also apply to RMT (40). Consequently, progressive overload, periodization, training specificity, and reversibility should be considered when designing RMT programs. To implement progressive overload in RMT, the workload must be gradually increased over time to stimulate adaptation. This can be achieved by progressively adjusting variables such as frequency, intensity, or duration of training sessions. For IPTL and TFRL, it is recommended to increase the resistance and maintain a similar number of training sessions or repetitions. Most of the protocols are based on 30 inspiratory maneuvers per session, and if these 30 maneuvers stop being a challenge, the resistance should be increased (29). For VIH, the progressive overload should be achieved by increased breathing frequency and total training time per week (41). When considering periodization, it is warranted to focus on efficient breathing patterns and proper RMT technique before adding moderate or high training loads. Another important aspect to consider is that RMT typically results in a plateau concerning maximum dynamic inspiratory muscle function after 6–9 weeks of training (40). Hence, to optimize RMT periodization, the training method could be adjusted every six to eight weeks. For example, this might involve transitioning from VIH to IPTL or alternating phases emphasizing lower repetitions with higher resistance and higher repetitions with lower resistance (42). During a detraining period, the respiratory muscle exhibits a decline in force-generation ability similar to that observed in limb muscles with similar practical implications (43). However, short periods without RMT should not result in significant functional gains. Notably, 8–12 weeks after RMT cessation, small yet significant declines in inspiratory muscle function were observed (40). Interestingly, reducing training frequency by 67% allowed for the maintenance of respiratory function during the observed 18 weeks, and even after discontinuing RMT the athletes exhibited improved pulmonary parameters compared with their pre-RMT values (40, 44).

Although employing RMT during exercise caught the attention of researchers and coaches, it is not a recommended combination (45). Additional respiratory loading during aerobic exercise leads to deterioration of performance due to an inadequate ventilatory response, breathing discomfort, anxiety, and intensification of effort (46). Rodrigues and McConnell (45) argued that additional RMT during exercise had the same pitfalls as training at high altitudes (45). Similarly, as the advantages of altitude training could be optimized by adopting the ‘live-high-train-low’ paradigm where benefits of altitude exposure are achieved without compromising training quality, separating RMT sessions and specific exercises is recommended (47).

RMT is generally considered a low-risk, safe activity when performed in accordance with the manufacturers’ guidelines. Some athletes, particularly women, may experience minor acute effects such as headaches or dizziness. The training load associated with RMT is small yet noticeable, therefore should be taken into account during training programming to limit the risk of overtraining or overreaching (41).

## Environmental and population factors

Although the presented guidelines are universal and may be applied in multiple settings, environment- and population-specific contexts should be considered. For example, exercising in hypoxia might constitute an additional challenge for the respiratory system and contribute to respiratory muscle fatigue due to increased work of breathing (48, 49). The use of hypoxic conditions in team sports is relatively limited, both in terms of altitude training and in preparation for competition at altitude, compared to the well-established practices in endurance sports (50). However, several team-sports arenas are situated at high altitude, including Mexico City's Estadio Azteca (2,200 m above sea level, ASL) and Estadio Akron in Guadalajara (1,672 m ASL), both of which will host matches during the 2026 FIFA World Cup. A recent review synthesizing findings from seven independent studies (investigating altitudes from 1,400 to 5,500 m ASL) highlighted the advantages of RMT for performance under hypoxic conditions (51). The outcomes revealed that RMT helped reduce fatigue in the respiratory muscle, enhanced the clearance and tolerance of anaerobic byproducts, postponed the activation of the respiratory muscle metaboreflex, and supported oxygen saturation and blood flow to the muscles involved in the movement (51). These multidimensional, positive influences are well-documented. However, the reviewed studies generally focused on incremental tests and performance-related physiological variables, rather than intermittent performance. Consequently, further research could explore how RMT affects RSA and YYT under various oxygen availability conditions.

Available research suggests that the respiratory system may impose greater limitations on athletic performance in women than in men (52). Compared to men of similar anthropometric indices, women generally have smaller lung volumes, reduced diffusion surface area, lower maximal expiratory flow rates, and narrower airways. As a result, they experience a higher work of breathing, greater airway hyperresponsiveness, more pronounced expiratory flow limitations, and an increased likelihood of exercise-induced arterial hypoxemia (53, 54). Consequently, RMT might be more beneficial in women in regular environments and prior to or during altitude exposure (55).

Scientific reporting on RMT and intermittent exercise performance in disabled team-sports athletes is scarce and not conclusive regarding best practices. Contrary to body-abled well-trained athletes, simple sport-specific training might have a significant positive influence on respiratory function (56). Also, it was suggested that combined inspiratory and expiratory training might be the most effective modality in athletes with spinal cord injuries (57). More specifically, studies on well-trained wheelchair basketball players present mixed results depending on the applied protocol. RMT of 12 weeks with progressive overload towards 70% of maximum inspiratory pressure increased YYT performance and maximal inspiratory strength recovery (58), whereas a shorter and lower-dose program (6 weeks and only 50% of maximum inspiratory pressure) did not result in improvement of RSA (59). However, even in the latter study, the athletes reported “less breathlessness” and “less tightness in the chest during the training”, and improved respiratory muscle function was observed (59). The differences may also be associated with the heterogeneity of the sample, which is a natural limitation of studies in Paralympic athletes (60). Overall, studies using cardiopulmonary exercise testing instead of intermittent exercise performance assessment to evaluate performance are prevalent in the disabled. Many report improved respiratory muscle strength (57), but the reports on exercise capacity are mixed (57, 61). The available evidence suggests that although RMT may improve pulmonary function, it should not be considered the primary method for improving the exercise performance of athletes with disabilities.

State-of-the-art research provides guidance regarding environments and populations discussed in this section. However, studies concerning RMT's influence on intermittent exercise performance in hypoxia, between-gender differences, or the disabled are scarce or non-existent. Consequently, any relevant coaching decisions would be an educated guess rather than following well-established, evidence-based protocol. Further research might not only address the abovementioned populations and environments, but also analyze less-investigated RMT methods such as TFRL or VIH.

## Conclusions and practical application

• Traditional sport-specific training or exercise programs lack sufficient stimulus to improve the function of the respiratory muscle, underscoring the rationale of integrating additional RMT into training regimens.
• RMT has the potential to improve intermittent exercise performance in team sports athletes, especially in women.
• Most of the interventions resulting in a significant, positive influence on intermittent exercise performance employed inspiratory pressure threshold loading, lasted 6–8 weeks, and relied on a high frequency of training sessions, progressive overload, and relatively high initial resistance (starting intensity).
• The respiratory muscle shares structural and functional similarities with other striated muscles, allowing standard training principles such as progressive overload, periodization, specificity, and reversibility to be applied when creating RMT programs.
• A variety of RMT devices and protocols can be tailored to the athlete's training level, preference, and performance goals. Common protocols may include performing 30 inspiratory maneuvers twice a day, five days a week, with resistance-based trainers or engaging in 20–40 min of vigorous ventilation with isocapnic devices every other day.
• Less-investigated RMT methods, such as TFRL or VIH, should be analyzed in the context of intermittent exercise performance. Moreover, further research addressing RMT and hypoxia, between-gender differences, and athletes with disabilities seems warranted.
